# Prevalence and risk factors for lung involvement on low-dose chest CT (LDCT) in a paucisymptomatic population of 247 patients affected by COVID-19

**DOI:** 10.1186/s13244-020-00939-7

**Published:** 2020-11-17

**Authors:** Maxime Castelli, Arnaud Maurin, Axel Bartoli, Michael Dassa, Baptiste Marchi, Julie Finance, Jean-Christophe Lagier, Matthieu Million, Philippe Parola, Philippe Brouqui, Didier Raoult, Sebastien Cortaredona, Alexis Jacquier, Jean-Yves Gaubert, Paul Habert

**Affiliations:** 1 Radiology Department, La Timone Hospital, Assistance Publique Des Hôpitaux de Marseille, 264 Rue Saint Pierre, 13005 Marseille 05, France; 2 UMR 7339, CNRS, CRMBM-CEMEREM (Centre de Résonance Magnétique Biologique et Médicale – Centre d’Exploration Métaboliques par Résonance Magnétique), Assistance Publique - Hôpitaux de Marseille, Aix-Marseille Université, 13385 Marseille, France; 3 IHU-Méditerranée Infection, Marseille, France; 4 IRD, APHM, Aix Marseille Univ, MEPHI, Marseille, France; 5 IRD, APHM, Aix Marseille Univ, VITROME, Marseille, SSA France; 6 LIIE, Aix Marseille Univ, Marseille, France; 7 CERIMED, Aix Marseille Univ, Marseille, France

**Keywords:** COVID-19, Tomography, X-ray computed, Quantitative evaluation, Pneumonia

## Abstract

**Background:**

Low-dose chest CT (LDCT) showed high sensitivity and ability to quantify lung involvement of COVID-19 pneumopathy. The aim of this study was to describe the prevalence and risk factors for lung involvement in 247 patients with a visual score and assess the prevalence of incidental findings.

**Methods:**

For 12 days in March 2020, 250 patients with RT-PCR positive tests and who underwent LDCT were prospectively included. Clinical and imaging findings were recorded. The extent of lung involvement was quantified using a score ranging from 0 to 40. A logistic regression model was used to explore factors associated with a score ≥ 10.

**Results:**

A total of 247 patients were analyzed; 138 (54%) showed lung involvement. The mean score was 4.5 ± 6.5, and the mean score for patients with lung involvement was 8.1 ± 6.8 [1–31]. The mean age was 43 ± 15 years, with 121 males (48%) and 17 asymptomatic patients (7%). Multivariate analysis showed that age > 54 years (odds ratio 4.4[2.0–9.6] *p* < 0.001) and diabetes (4.7[1.0–22.1] *p* = 0.049) were risk factors for a score ≥ 10. Multivariate analysis including symptoms showed that only age > 54 years (4.1[1.7–10.0] *p* = 0.002) was a risk factor for a score ≥ 10. Rhinitis (0.3[0.1–0.7] *p* = 0.005) and anosmia (0.3[0.1–0.9] *p* = 0.043) were protective against lung involvement. Incidental imaging findings were found in 19% of patients, with a need for follow-up in 0.6%.

**Conclusion:**

The prevalence of lung involvement was 54% in a predominantly paucisymptomatic population. Age ≥ 55 years and diabetes were risk factors for significant parenchymal lung involvement. Rhinitis and anosmia were protective against LDCT abnormalities.

## Background

In December 2019, a series of pneumonia cases caused by a novel coronavirus occurred in Wuhan, Hubei, China [1–3]. The severe acute respiratory syndrome coronavirus 2 (SARS-CoV-2) was named by the International Committee on Taxonomy of Viruses [4]. Coronavirus disease 2019 (COVID-19) spread worldwide from Asia to Europe and is now in the ascending phase of the epidemic in America [2]. The World Health Organization (WHO) declared it a world pandemic situation on March 11, with over 110,000 cases, and the number is still increasing [5].

Involvement of the disease has a wide variety of clinical features, from cough to pulmonary failure [6, 7]. Moreover, a large number of patients remain totally asymptomatic, allowing the pandemic to spread even more easily [8, 9]. Reverse -transcription polymerase chain reaction (RT-PCR) is the main tool for diagnosis but does not allow for the assessment of disease severity. Chest X-ray is not recommended in the initial phase of the disease due to its low value to detect ground-glass opacities. Low-dose chest computed tomography (LDCT) appears to be a useful tool in the management of patients during the COVID-19 epidemic. LDCT is very sensitive for diagnosis, quantification of disease severity and identification of a differential diagnosis. Furthermore, after recovery, LDCT might be of interest in the prediction of lung fibrosis during healing [10–13]. Kang et al. emphasized the role of LDCT in the diagnosis of COVID-19, especially in the early stages of the disease. Furthermore, Burian et al. showed that the proportion of lung involvement could be a risk factor for hospitalization in the intensive care unit, suggesting that the extent of lung involvement is clinically relevant [14]. The goal of the study was to (1) determine the prevalence and risk factors for lung involvement on LDCT according to clinical symptoms and comorbidities in all consecutive patients with a positive RT-PCR test over a short period of time (12 days) and (2) evaluate whether LDCT is able to detect other abnormalities in an incidental way that requires care or just medical follow-up.

## Methods

### Study design

This was a single-centre prospective study conducted from the 18th to the 30th of March 2020. Patient enrolment included all consecutive patients presenting to the department of infectious disease for 12 consecutive days with a diagnosis of COVID-19 confirmed by RT-PCR [15] who underwent an unenhanced chest CT with a low-dose protocol. All LDCT procedures were performed at least 24 h after the RT-PCR test, and patients only underwent LDCT in cases of positive results on nasal swabs. Virological diagnosis of SARS-CoV-2 infection was performed using sample nasopharyngeal swabs with a hydrolysis probe-based real-time reverse transcription-PCR system that targets the envelope (E) protein-encoding gene [15, 16]. LDCT was performed to describe the type and prevalence of lung involvement. The exclusion criteria were LDCT scan refusal. The protocol was approved by the local institutional review board.

### Clinical data

For each patient relevant clinical data were recorded by the infectiologist the same day before LDCT, the following clinical parameters were recorded: age, sex, date of the first symptoms, temperature, heart rate, systolic and diastolic blood pressure, respiratory rate, oxygen saturation, cough, rhinorrhea, dyspnea, diarrhea, myalgia, and lung auscultation abnormalities. The national early warning score (NEWS) was rated. Symptoms were recorded as present or absent, dyspnea was defined as the feeling of shortness breath, diarrhea was defined by liquid tools more than 3 times a day, myalgia was defined as muscles aches without recent intensive sport practice, and lung auscultation abnormalities as crackling in a focal part of the lungs. Medical history was recorded: heart disease, tobacco use, chronic obstructive pulmonary disease (COPD), asthma, diabetes, obesity, sleep apnea syndrome, oncologic status and immunosuppression status. The delay between the first symptoms and the LDCT was classified as < 4 days, 4–7 days, 8–11 days and > 11 days.

### LDCT

All patients underwent LDCT on the same system (Revolution EVO-GE Healthcare, WI, USA). All LDCT unenhanced scans were acquired in profound and maximal inspiration with the following parameters: detector collimation: 0.625 mm; field of view: 500 mm; matrix: 512 × 512; pitch: 1.375; gantry speed 0.35 s; 120 kV; 45 mAs; and reconstructed slice thickness 1.2 mm. All imaging data were reconstructed using high resolution and standard algorithms. LDCT data were sent directly to a picture archiving and communicating system. Monitors were used to view both mediastinal (width, 400 HU; level, 20 HU) and lung (width, 1600 HU; level, -600 HU) windows. The pre-established top anatomic border was the lower part of the neck. The pre-established anatomic bottom boundary was the estimated location of the adrenal glands below the costophrenic angle. LDCT data were sent to an archiving and communicating system (PACS) (Centricity Universal Viewer – GE Healthcare, WI, USA).

LDCT scans were analyzed by two thoracic radiologists with more than 25 and 7 years of experience (JYG and PH). Imaging was reviewed independently and final decisions were reached by consensus. For each patient, the delay between the first symptoms and the date of chest CT was recorded. Abnormalities were described according to the Fleischner glossary [17]. The features encountered during the disease were described as exclusive ground-glass opacities (GGO) crazy paving patterns which is GGO and septal thickening in the same area, areas of consolidation, pleural effusion, peribronchovascular thickening, and mediastinal and hilar nodes. We also reported all incidental imaging findings, meaning abnormalities that were not related to COVID-19.

### CT-scan severity score (CT-SS)

**Fig. 1:**
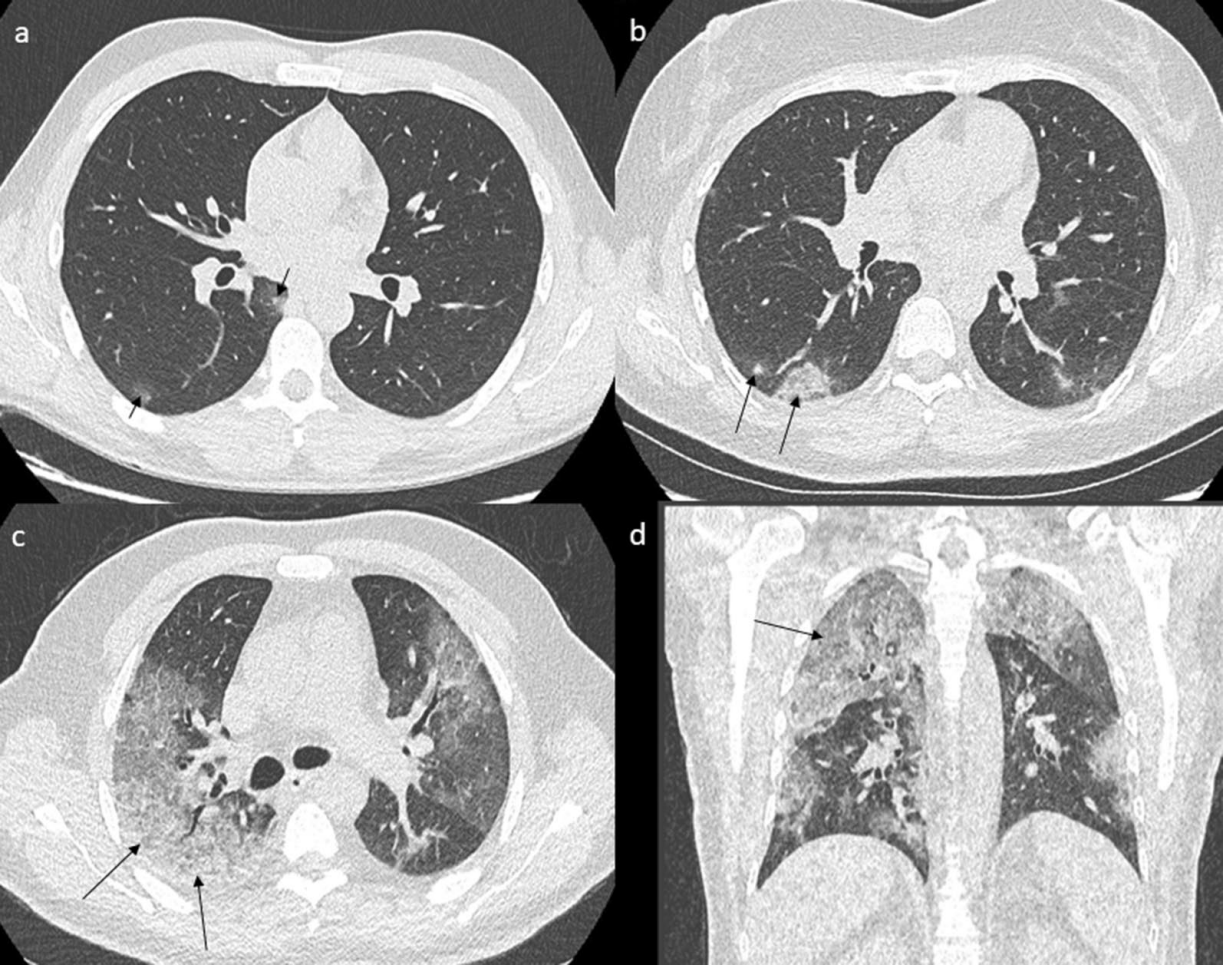
CT-SS, instances of different involvements. Note: **a** A 27-year-old male COVID-19 patient with no medical history presenting cough, anosmia and fever for 4 days. LDCT shows two GGOs in the right 6th segment (arrow) corresponding to a minimal impairment with the presence of < 10 secondary lobules. The global CT-SS was 1. **b** A 44-year-old female COVID-19 patient with no medical history presenting cough, rhinorrhea and myalgia for 5 days. LDCT shows GGO in the right 6th segment (arrow) corresponding to an intermediate impairment with the presence of < 50% involvement of the 6th segment. The global CT-SS was 4. **c**, **d** A 65-year-old male COVID-19 patient with diabetes and hypertension presenting cough, rhinorrhea, anosmia, myalgia and dyspnea for 7 days. LDCT shows GGO and partial consolidation in the right 2nd segment (arrows), corresponding to severe impairment with more than 50% involvement of the segment. The global CT-SS was 30

CT-SS was used to quantify the extent of lung abnormalities [18]. It was obtained by adding the notes attributed to each lung segment [19]. The extent of the lesions (GGO, crazy paving or areas of consolidation) was visually classified into 3 different types for each segment: lack of lesion, intermediate involvement and severe involvement. Lack of involvement was defined as a strictly normal pattern and was rated 0. Involvement was considered intermediate for a segment with less than 50% involvement and was rated 1. When involvement was more than 50% of a segment, it was defined as severe and rated 2. The final CT-SS was obtained by summing the score of each segment, to reflect the number of segments which correctly performed the hematosis. It was ranked between 0 (no lesion) and 40 (all right and left segments with more than 50% involvement) (Fig. 1).

### Statistics

Continuous and categorical variables are presented as the mean (SD), range and *n* (%), respectively. We used the Mann–Whitney and Kruskal–Wallis tests to compare the score values. To explore risk factors associated with a score greater than or equal to 10, we also performed multivariable analyses using a logistic regression model. A two-sided *α* of less than 0.05 was considered statistically significant. All analyses were carried out using SAS 9.4 statistical software (SAS Institute, Cary, NC).

## Results

### Population

Two hundred and fifty patients with a positive RT-PCR test between the 18th and 30th of March were consecutively selected for analyses, and all data were available for 247/250 patients (99%). The mean age of the population was 43 years ± 15 with a minimal age of 18 years and a maximum age of 83 years, with 117 males (47%) and 130 females (53%). The distribution of patients according to age was 3 (1%), 82 (33%), 47 (19%), 46 (19%), 47 (19%), 46 (19%) and 22 (9%) for classes < 18 years, 18–34 years, 35–44 years, 45–54 years, 55–64 years and more than 65 years, respectively. Included patients were tested using nasal swabs if they had symptoms or if they were in close contact with an affected patient (Table 1).

**Table 1:** Clinical and LDCT data (*n* = 247)

Clinical data	All (*n* = 247)
Age (years)—mean ± SD	43 ± 15 [18–86]
Sex (male)	117/247 (47%)
Symptoms	230/247 (93%)
Cough	131/247 (53%)
Rhinitis	94/247 (38%)
Fever	40/247 (16%)
Anosmia	110/247 (45%)
Ageusia	102/247 (41%)
Dyspnea	68/247 (28%)
Chest pain	44/247 (18%)
Needed oxygen	7/247 (3%)
Desaturation	27/247 (11%)
Hospitalization	9/247 (4%)
Delay between first symptoms and chest CT (days)—mean ± SD	8 ± 4 [0–23]
Diabetes	12/247 (5%)
Hypertension	27/247 (11%)
Cancer status	9/247 (4%)
Cardiac disease	7/247 (3%)
LDCT data	
Dose-length product (mGy cm)—mean ± SD	39.8 ± 6.3
Global score-mean ± SD	4.5 ± 6.5
Normal LDCT	109/247 (44%)
Exclusive peripheral abnormalities	98/247 (40%)
Exclusive GGOs	102/247 (41%)
Crazy paving pattern (GGOs + septal thickening)	16/247 (6%)
GGOs + areas of consolidation	30/247 (12%)

Clinical and LDCT data in the whole population. The qualitative variables are expressed as figures with percentages, and the continuous variables are expressed as the mean value ± DS

*LDCT* low-dose computed tomography

### Clinical data

Most patients (230/247, 93%) sought medical consultation because they suffered from at least one symptom, and 17/247 patients (7%) were asymptomatic. The most common symptoms were cough for 131/247 (53%), anosmia for 110/247 (45%), rhinitis for 94/247 (38%), ageusia for 102/247 (41%), dyspnea for 68/247 (28%), and thoracic pain for 44/247 (18%). The most frequently observed medical histories were high blood pressure: 27/247 (11%), chronic pulmonary diseases (including asthma, COPD and sleep apnea syndrome): 25/247 (10%), and diabetes: 12/250 (5%). Other medical histories were both cancer and immunosuppression for 9/247 (4%) and coronary heart disease for 7/247 (3%). At the first examination, before LDCT, fever (central temperature ≥ 38 °C) was observed in 40/247 patients (16%). Oxygen desaturation (Sa0_2_ < 95%) without oxygen supply was observed in 27/247 patients (11%), oxygen was needed for 7/247 patients (3%), and hospitalization was required for 9/247 (4%). Due to the low rate of hospitalization and only ambulatory care for 96% of patients, we defined this population as predominantly paucisymptomatic (Table 1).

### LDCT data and CT-SS

**Fig. 2:**
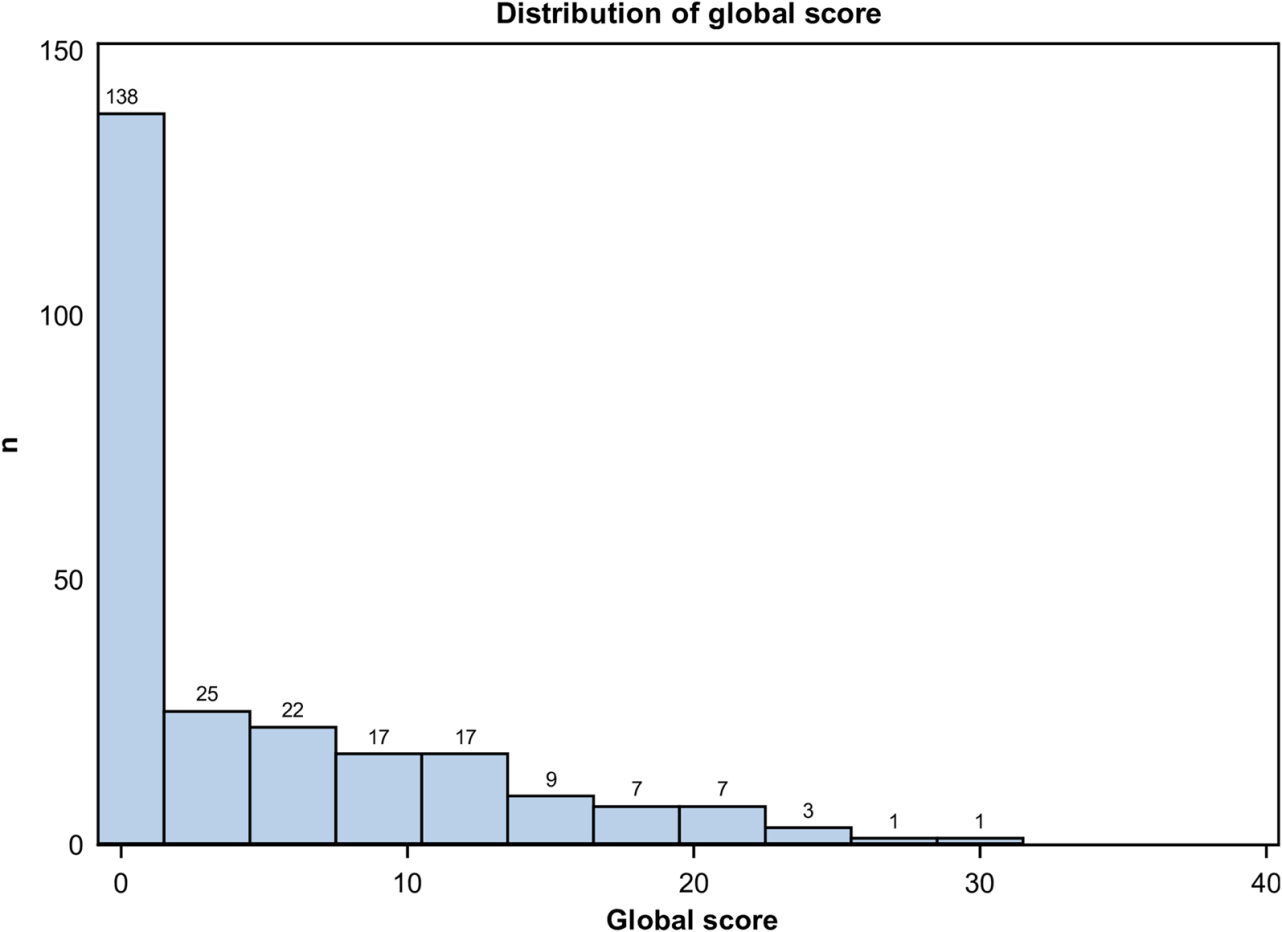
Distribution of global score ranking from 0 to 40 in the 247 patients studied. Note: This was a paucisymptomatic population, and the majority of patients had a score lower than or equal to 10

All chest CT scans were performed with a low-dose protocol, and the mean dose-length product (DLP) was 39.8 mGy cm ± 6.3. The mean delay between the first symptoms and the chest CT was 8 days ± 4. The mean and standard deviation of the CT-SS in the whole population was 4.5 ± 6.5, and 51/247 (21%) had an CT-SS ≥ 10 (Fig. 2). A total of 109/247 chest CTs (44%) were normal, without any features of COVID-19 pneumonia. Consequently, their score was 0/40. A total of 138/247 chest CTs (56%) were abnormal. Their mean CT-SS was 8.1 ± 6.8. Among them, 87/138 chest CTs (63%) presented a score between 1 and 9, and 51/138 patients (37%) presented a score ≥ 10. A total of 98/247 patients (40%) presented exclusively with peripheral lesions regardless of the lesion. 104/247 (42%) presented exclusively GGOs lesions. Features such as GGOs and areas of consolidation were associated in 32/247 patients (33%). 16/247 patients (16%) demonstrated only crazy paving patterns. Pleural effusion was seen in 2 patients (2%) (Table 1).

One hundred thirty-seven patients had NEWS = 0 (55%) and 59/137 (57%) patients had abnormalities on LDCT. Their mean score was 2.3 ranking from 0 to 17. There was a significant difference with patient which had a NEWS > 0; their mean score was 7.3 ranking from 0 to 31 (*p* < 0.001).

**Fig. 3:**
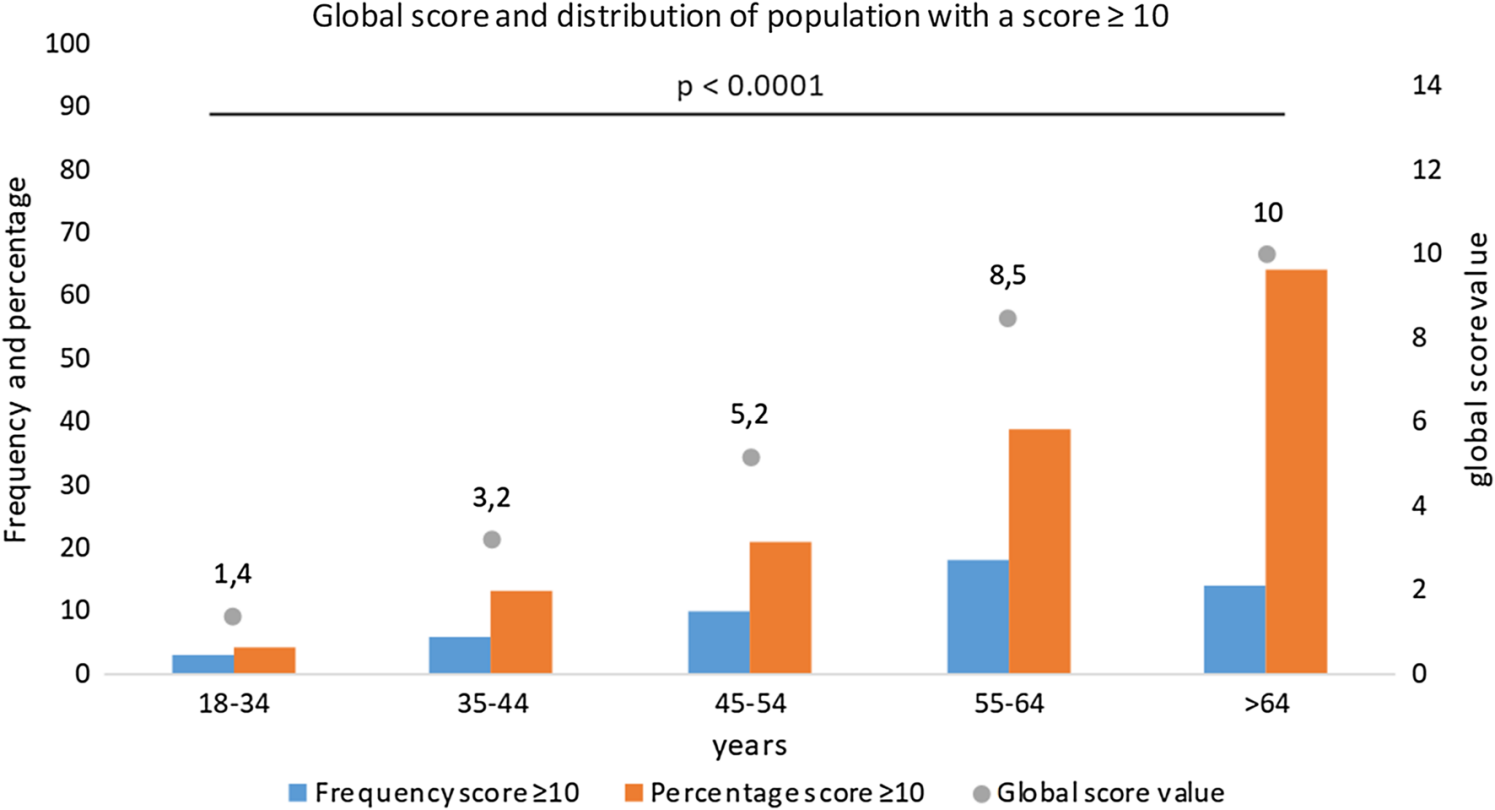
Global score and distribution of population with a score ≥ 10 across age. Note: We can see that the percentage of the population with a score ≥ 10 and the global score is higher when patients are older. There was a significant difference between all groups

**Fig. 4:**
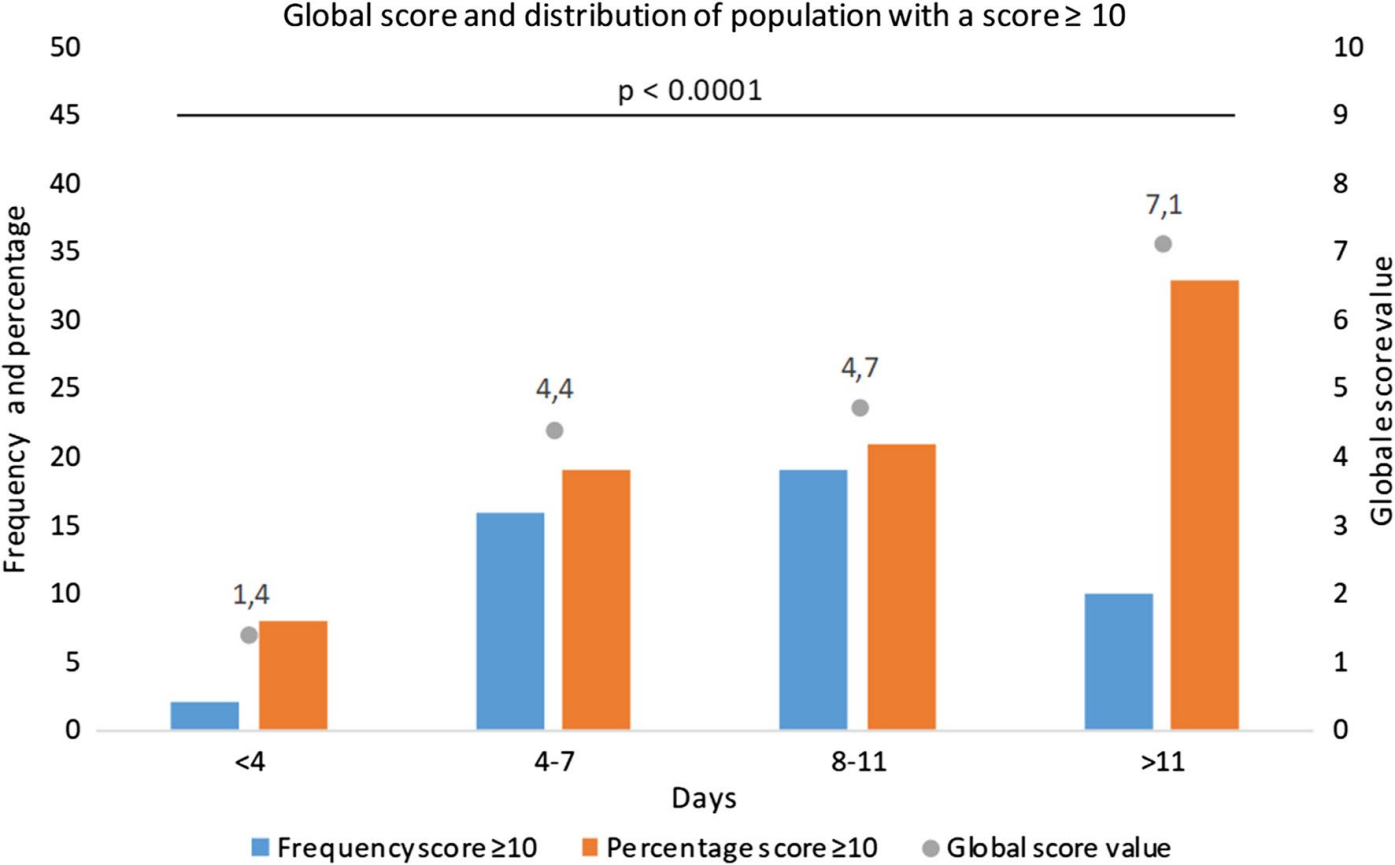
Distribution of the population with a score ≥ 10 and mean score according to delay between symptom appraisal and chest CT. Note: We can see that the global score is higher and the proportion of score ≥ 10 is also higher according to the delay between the first symptom occurring and the chest CT. There was a significant difference between all groups

The CT-SS was not statistically different according to sex or the presence of symptoms, whereas the score was significantly higher if fever (6.9 ± 6.2 versus 4.1 ± 6.4; *p* = 0.002) or desaturation were noted (13.2 ± 7.8 versus 3.5 ± 5.4; *p* < 0.001), interestingly the score was also higher in patient that will required hospitalization (15.3 ± 8.4 versus 4.1 ± 6.0; *p* < 0.001) (Table 2). The CT-SS increased significantly according to age as well as the delay between first symptoms and LDCT (Figs. 3, 4).

**Table 2:** CT-SS according to clinical data (*n* = 247)

	COVID-19 LDCT		*p* value
	Score		
	Mean ± SD		
	No	Yes	
Clinical data			
Sex (male/female)	5.1 ± 6.9	4.0 ± 6.0	0.247
Symptoms	4.3 ± 6.4	4.5 ± 6.5	0.834
Rhinitis	4.9 ± 6.7	3.9 ± 6.1	0.223
Fever	4.1 ± 6.4	6.9 ± 6.2	**0.002**
Anosmia	5.2 ± 7.3	3.7 ± 5.2	0.342
Ageusia	4.6 ± 6.8	4.4 ± 6.0	0.805
Dyspnea	3.5 ± 5.3	7.2 ± 8.3	**< 0.001**
Chest pain	4.6 ± 6.7	4.0 ± 5.1	0.89
Needed oxygen	4.0 ± 5.8	20.9 ± 7.9	**< 0.001**
Desaturation	3.5 ± 5.4	13.2 ± 7.8	**< 0.001**
Hospitalization	4.1 ± 6.0	15.3 ± 8.4	**< 0.001**
Hypertension	3.9 ± 5.9	10.0 ± 8.1	**< 0.001**
Diabetes	4.1 ± 6.2	12.0 ± 7.3	**< 0.001**
Cancer status	4.3 ± 6.3	11.3 ± 7.0	**0.003**
Cardiac disease	4.4 ± 6.4	9.1 ± 7.2	**0.043**
Demographics data			
Age 18–34 years	1.4 ± 2.8		**< 0.001**
Age 35–44 years	3.2 ± 5.0		
Age 45–54 years	5.2 ± 5.6		
Age 55–64 years	8.5 ± 9.1		
Age > 64 years	10.0 ± 6.9		
Delay between symptoms and LDCT			
0–3 days	1.4 ± 3.6		**0.003**
4–7 days	4.4 ± 6.8		
8–11 days	4.7 ± 6.2		
> 11 days	7.1 ± 7.3		

All significant results with a *p* < 0.05 have been highlighted in bold

Distribution of the CT-SS according to symptoms across age and delay between symptoms and LDCT. The continuous variables are presented as the mean value ± DS

*COVID-19* coronavirus disease 2019, *LDCT* low-dose computed tomography, *p* value: Wilcoxon Mann–Whitney/Kruskal–Wallis test

If patients were under 55 years old, with no dyspnoea, no desaturation and no comorbidities (hypertension, diabetes, heart disease and cancer history), the score was always less than 10.

In a multivariate analysis, two models were used, the first only on comorbidities, and the odds ratio (OR) of having a score ≥ 10 was 4.4 ([2.0–9.6] *p* < 0.001) for age > 54 years; the OR was 4.7 ([1.0–22.1] *p* = 0.049) for diabetes, 1.8 ([0.6–4.9] *p* = 0.265) for hypertension, and 4.5 ([0.7–28.4] *p* = 0.106) for cancer status (Table 3). In the second model including symptoms and comorbidities, only age was a risk factor for a score > 10 with an OR equal to 4.1 ([1.7–10.0] *p* = 0.002). No symptoms were risk factors for an LDCT score ≥ 10, but rhinitis and anosmia were protective, with ORs of 0.3 ([0.1–0.7] *p* = 0.005) and 0.3 ([0.1–0.9] *p* = 0.043), respectively (Table 4).

**Table 3:** Comorbidities associated with CT-SS ≥ 10—multivariate logistic regression (*n* = 247)

Covariate	OR 95% CI	*p* value
Age > 54 years	4.4 [2.0–9.6]	**< 0.001**
Diabetes	4.7 [1.0–22.1]	**0.049**
Hypertension	1.8 [0.6–4.9]	0.265
Cancer status	4.5 [0.7–28.4]	0.106
Cardiac heart disease	2.1 [0.3–14.3]	0.455

All significant results with a *p* < 0.05 have been highlighted in bold

Multivariate analysis to determine which clinical factors or comorbidities are predictive of CT-SS ≥ 10

* LDCT* low-dose chest CT, *OR 95% CI* odds ratio with 95% confidence interval

**Table 4:** Comorbidities and symptoms associated with CT-SS ≥ 10—multivariate logistic regression (*n* = 247)

Covariate	OR 95% CI	*p* value
Age > 54 years	4.1 [1.7–10.0]	**0.002**
Cough	2.3 [0.9–5.5]	0.063
Rhinitis	0.3 [0.1–0.7]	**0.005**
Fever	1.9 [0.8–4.9]	0.169
Anosmia	0.3 [0.1–0.9]	**0.043**
Ageusia	2.2 [0.8–6.6]	0.150
Dyspnea	2.2 [0.9–5.1]	0.070
Chest pain	0.7 [0.3–2.1]	0.574
Diabetes	4.6 [0.9–23.3]	0.063
Hypertension	2.2 [0.7–7.1]	0.196
Cancer status	0.7 [0.1–9.8]	0.079
Cardiac heart disease	2.0 [0.3–15.5]	0.483

All significant results with a *p* < 0.05 have been highlighted in bold

Multivariate analysis to determine which clinical factors or comorbidities are predictive of CT-SS ≥ 10

*LDCT* low-dose chest CT, *OR 95% CI* odds ratio with 95% confidence interval

### Incidental findings

Incidental imaging findings were found in 47 patients (19%), 5 patients had 2 incidental findings (2%), and 1 presented 3 incidental findings (1%). An aneurismal dilatation of the ascending aorta between 40 and 50 mm in diameter was found in 5 patients (2%). Pulmonary arteriovenous malformation, thymic tumor and bronchogenic cyst were found in 1 patient (0.4%). Steatosis was found in 21 patients (8.4%). Two patients (0.8%) had undetermined lesions on the liver. Asymptomatic gallstones were found in 2 patients (0.8%). Adrenal adenomas were found in 4 patients (1.6%). One patient (0.4%) had an undetermined lesion on a kidney. Urinary stones were found in 5 patients (2%). Splenomegaly, unilateral gynecomastia, and abdominal nodes were each found in 1 patient (0.4%). At least one vertebral fracture was found in 3 patients (1%). Three incidental pulmonary nodules (1.2%) were found that needed follow-up. Among all these findings, only 14/247 (0.6%) required follow-up.

## Discussion

In a predominantly paucisymptomatic patient cohort of 247 consecutive COVID-19 patients, we found a prevalence of 138/247 (56%) patients with lung involvement. A total of 109/247 (46%) had a normal CT. Risk factors for lung involvement were age > 54 years and diabetes in multivariate analysis on comorbidities and only age > 54 years in multivariate analysis including comorbidities and symptoms. Rhinitis and anosmia appeared to be protective factors against lung involvement. Incidental findings were noticed in 47/247 patients (19%), but only 14/247 (0.6%) needed follow-up.

These results show that age > 54 years and diabetes were risk factors for finding at least moderate lung involvement on LDCT, which is in line with other reported data about comorbidities on COVID-19 patients’ risk [20, 21]. A meta-analysis of 1558 patients found that significant risk factors were hypertension, diabetes, chronic obstructive pulmonary disease, cardiovascular disease, and cerebrovascular disease. Another Italian retrospective study from Grasselli et al. on 1591 patients in the intensive care unit showed that the mortality rate was superior in patients with ages greater than 64 years compared with younger patients [22, 23]. Since no clinical symptoms were risk factors for severe impairment on LDCT, probability to have an abnormal chest CT should be reliably based on comorbidities rather than symptoms. Another study on mortality likewise showed that no symptoms were risk factors for mortality [24] that is consistent with no symptoms were risk factor to find abnormalities on LDCT. This prevalence is in line with previous study on paucisymptomatic population, asymptomatic cases from the cruise ship “Diamond Princess” were 73%, with 54% of them showing lung abnormalities on CT, whereas 27% were symptomatic and 79% of them had abnormal findings on CT [25]. Yang et al. in a letter to the editor explained that the prevalence of pneumonia on CT is very different according to different study and he recall that prevalence rely on the population explored, a significant number of paucisymptomatic patient might have lung involvement [26].

Initial LDCT in paucisymptomatic cases could be helpful to better assess fibrosis development after recovery. After SARS-CoV-1 infection, a study showed that fibrosis features on CT more than 4 weeks after initial symptoms included reticular opacities, architectural distortion and bronchial dilatation in the same area as initial involvement [27]. More than 4 months after recovery, approximately 10% of 258 SARS-CoV-1 patients had permanent sequelae, especially older patients and those who required intensive care units for acute distress respiratory syndrome [28].

A pitfall of LDCT is the absence of contrast injection. COVID-19 disease has been shown to be a risk factor for pulmonary embolism [29] especially in the intensive care unit [30]. During data analysis, there was no evidence of a link between COVID-19 and the risk of thrombosis. The population studied was predominantly a paucisymptomatic population. Initially, D-dimers were not among biological dosages, and most of the patients had a low risk for thrombosis according to usual scores such as Wells or modified Geneva score. Following the evidences of recent publications, D-dimers have been systematically tested, and if the result is positive, chest CT angiography is performed instead of LDCT [31]. Currently, during follow-up, in cases of clinical worsening, unexplained tachycardia or persistent inflammatory syndrome, chest CT angiography is performed to ensure the absence of pulmonary embolism.

The published CT-SS has been chosen to classify disease extension on LDCT because this is a visual score permitting the quantification of lung involvement [18]. These advantages are that this method is easy to use, quick, wide and gradual, ranging from 0 to 40, and can differentiate mild from severe cases. Many scores have been suggested using quantitative or qualitative indicators [32, 33]. The CT score from Francone et al. has shown that a high score > 17/25 is highly predictive of patient mortality in short-term follow-up and that the parenchyma assessment is more accurate than inflammatory biomarkers in a multivariate analysis to evaluate the short-term outcome. A CO-RADS score was developed by Prokop et al. in the same way as TI-RADS or BI-RADS, but for suspected COVID-19 disease than to quantify the extent of the disease. In the presented population, due to positive RT-PCR results for all 247 patients, LDCT patients were all CO-RADS 6 [34].

Regarding the interest of the use of LDCT in COVID-19, a low-dose protocol has been recently recommended [35], but no significant results in routine clinical practice have been published to date. Since many of the patients should be referred for follow-up, LDCT is probably the best option, especially for younger patients [36]. LDCT may be repeated when clinical worsening occurs to detect a pneumothorax or if an additional bacterial infection occurs to look for lung abscess. This approach would ideal for patients in ambulatory care with a negative result for RT-PCR but with a high clinical suspicion for COVID-19 pneumonia to identify false negative nasal swab tests. Some studies have evaluated that a patient affected by COVID-19 could have 6 LDCTs during the course of the disease, and non-COVID-19 individuals could have 2 LDCTs to ensure that they are not affected [37].

To compare imaging features and prevalence of lung involvement for other viruses, such as influenza, parainfluenza and respiratory syncytial virus have been explored in symptomatic populations. The features encountered in these viruses are similar to those found for COVID-19 pneumonia. In a literature review, GGO was systematically associated with influenza lung involvement [38]. Several papers showed that the prevalence of lung abnormalities on chest CT was between 57 and 65% when pooling the data for all different viruses in a symptomatic population [39, 40]. In the presented population, the prevalence of abnormal findings was 54%, which could be considered high in paucisymptomatic patients. These results are very close to those found with severe acute respiratory syndrome coronavirus 1 (SARS-CoV-1) [41].

We reported incidental imaging findings in 47 patients (19%) and 14 patients (0.6%) will require follow-up. This low rate might be explained because our population is mostly young and has few comorbidities.

One limitation of this study is the short delay we observed in some of our patients between the first symptoms and LDCT, since chest CT may be normal in patients with COVID-19 within the 3 days following the appraisal of symptoms [42]. However, this percentage of patients was low in our population (25/247, 11%).

## Conclusion

In this population of predominantly paucisymptomatic COVID-19 patients, the prevalence of lung involvement was 54%. Neither clinical symptoms nor signs were predictors of lung involvement, but age > 54 years and diabetes were risk factors for having a CT-SS ≥ 10. Rhinitis and anosmia appeared to protect against lung involvement. Longer follow-up is required to define what type of lesion or which patients might evolve towards pulmonary fibrosis sequelae.

## Data Availability

Data are available.

## Abbreviations

COPD: Chronic obstructive pulmonary disease
COVID19: Coronavirus disease 2019
CT-SS: Computed tomography severity score
GGO: Ground-glass opacity
LDCT: Low-dose chest CT
NEWS: National early warning score
RT-PCR: Real-time reverse transcription-polymerase chain reaction
SARS-CoV-1: Severe acute respiratory syndrome coronavirus 1
SARS-CoV-2: Severe acute respiratory syndrome coronavirus 2
WHO: World Health Organization
