# Correction to: Prevalence and risk factors for lung involvement on low-dose chest CT (LDCT) in a paucisymptomatic population of 247 patients affected by COVID-19

**DOI:** 10.1186/s13244-020-00950-y

**Published:** 2021-02-16

**Authors:** Maxime Castelli, Arnaud Maurin, Axel Bartoli, Michael Dassa, Baptiste Marchi, Julie Finance, Jean-Christophe Lagier, Matthieu Million, Philippe Parola, Philippe Brouqui, Didier Raoult, Sebastien Cortaredona, Alexis Jacquier, Jean-Yves Gaubert, Paul Habert

**Affiliations:** 1 Radiology Department, La Timone Hospital, Assistance Publique Des Hopitaux de Marseille, 264 Rue Saint Pierre, 13005 Marseille 05, France; 2 UMR 7339, CNRS, CRMBM‑CEMEREM (Centre de Resonance Magnetique Biologique et Medicale – Centre d’Exploration Metaboliques par Resonance Magnetique), Assistance Publique - Hopitaux de Marseille, Aix-Marseille Universite, 13385 Marseille, France; 3 IHU-Mediterranee Infection, Marseille, France; 4 IRD, APHM, Aix Marseille Univ, MEPHI, Marseille, France; 5 IRD, APHM, Aix Marseille Univ, VITROME, Marseille, SSA France; 6 LIIE, Aix Marseille Univ, Marseille, France; 7 CERIMED, Aix Marseille Univ, Marseille, France

## Correction to: Insights Imaging (2020) 11:117 10.1186/s13244-020-00939-7

The original article [1] mistakenly omitted the Key Points section at the beginning due to an error by the production team that processed the article.

Ahead is the Key Points section as originally intended for presentation in the article:

## Key points

1. In a paucisymptomatic population the prevalence of COVID-19 pneumonia on LDCT was 54%.
2. Age > 54 years and diabetes were significant risk factors to detect score ≥ 10 in the multivariate analysis. Rhinitis and anosmia were protector.
3. We reported incidental imaging findings in 47 patients (19%) and 14 patients (0.6%) will require follow-up.

## References

[1] Castelli M, Maurin A, Bartoli A (2020). Prevalence and risk factors for lung involvement on low-dose chest CT (LDCT) in a paucisymptomatic population of 247 patients affected by COVID-19. Insights Imaging.

