# Author Correction: Prevalence and risk factors for lung involvement on low-dose chest CT (LDCT) in a paucisymptomatic population of 247 patients affected by COVID-19

**DOI:** 10.1186/s13244-024-01847-w

**Published:** 2024-11-08

**Authors:** Maxime Castelli, Arnaud Maurin, Axel Bartoli, Michael Dassa, Baptiste Marchi, Julie Finance, Jean‑Christophe Lagier, Matthieu Million, Philippe Parola, Philippe Brouqui, Didier Raoult, Sebastien Cortaredona, Alexis Jacquier, Jean‑Yves Gaubert, Paul Habert

**Affiliations:** 1 Radiology Department, La Timone Hospital, Assistance Publique Des Hopitaux de Marseille, 264 Rue Saint Pierre, 13005 Marseille 05, France; 2 UMR 7339, CNRS, CRMBM‑CEMEREM (Centre de Resonance Magnetique Biologique et Medicale – Centre d’Exploration Metaboliques par Resonance Magnetique), Assistance Publique ‑ Hopitaux de Marseille, Aix-Marseille Universite, 13385 Marseille, France; 3 IHU-Mediterranee Infection, Marseille, France; 4 IRD, APHM, Aix Marseille Univ, MEPHI, Marseille, France; 5 IRD, APHM, Aix Marseille Univ, VITROME, Marseille, SSA France; 6 LIIE, Aix Marseille Univ, Marseille, France; 7 CERIMED, Aix Marseille Univ, Marseille, France

**Correction to: Insights into Imaging**

10.1186/s13244-020-00939-7, published online 17 November 2020

The original article mistakenly referred to the study as “prospective” when, in fact, it was a retrospective study.

Following an investigation by the publisher and the *Insights into Imaging* Editorial Office that included contact with the author group and the person responsible for promoting trials at the Assistance Publique des Hôpitaux de Marseille, it was confirmed that the study was retrospective, and a copy of a research protocol was provided.

A review of this protocol found the timetable, focus, and methods listed to match the work, reported to have taken place between March and June 2020, attempting to identify predictors for COVID-19 patients; the methods specifically mention low-dose scans. The protocol describes the work as retrospective, with the data source listed as medical records. Although the reference number of this protocol is not mentioned by the authors in the article, it does match the reference methodology that the authors suggested be included in their original request for a correction.

The references to this as a prospective study have been changed to clarify the retrospective nature of this work.

The original article has been corrected.

